# Cervical Carcinoma: Oncobiology and Biomarkers

**DOI:** 10.3390/ijms222212571

**Published:** 2021-11-22

**Authors:** Larisa V. Volkova, Alexander I. Pashov, Nadezhda N. Omelchuk

**Affiliations:** 1 Laboratory of Immunohistochemistry, Pathology and Clinical Diagnostics, Department of Fundamental Medicine, Medical Institute, Immanuel Kant Baltic Federal University, 236041 Kaliningrad, Russia; 2 Department of Obstetrics and Gynecology, Medical Institute, Immanuel Kant Baltic Federal, 236041 Kaliningrad, Russia; Pachov@mail.ru; 3 Department of Clinical Laboratory Diagnostics, Faculty of Continuing Medical Education, People’s Friendship University, 117198 Moscow, Russia; kkld-fpkmr-nom@mail.ru

**Keywords:** carcinoma of the cervix, cervical carcinogenesis, biomarkers, immunohistochemistry

## Abstract

Cervical cancer is one of the most common types of carcinomas causing morbidity and mortality in women in all countries of the world. At the moment, the oncology, oncobiology, and oncomorphology of cervical cancer are characterized by the accumulation of new information; various molecular biological, genetic, and immunohistochemical methods of investigation of the mechanisms of cervical carcinogenesis are tested and applied; targeted antitumour drugs and diagnostic, prognostic, and predictive biomarkers are being searched for. Many issues of the etiopathogenesis of cervical cancer have not been sufficiently studied, and the role of many biomarkers characterizing various stages of cervical carcinogenesis remains unclear. Therefore, the target of this review is to systematize and understand several problems in the pathogenesis of cervical cancer and to evaluate the significance and role of biomarkers in cervical carcinogenesis.

## 1. Introduction

In the structure of morbidity and mortality, cervical cancer (CC) is one of the most common types of carcinomas among women in all countries of the world, in fourth place after breast cancer, colorectal cancer, and lung cancer [1]. According to the data of the International Agency for Research on Cancer, in 2018, the number of patients with cervical cancer was 570,000, an increase of 7.8% over the past decade, and the number of deaths from this disease amounted to 311,000 [1,2]. In 2020, 604,100 new cases of cervical cancer were detected around the world, and the number of deaths was 341,800 [1,2,3,4]. Therefore, despite the recommended screening programs for cervical cancer, morbidity and mortality rates from this oncopathology have been steadily growing in recent years, both worldwide and within the Russian Federation [4,5,6,7]. Indicators of later diagnosis of cervical cancer in stages III-IV of the disease in Russia have also reached high numbers—they made up 32.6% in 2018 [4]. Currently, the oncology, oncobiology, and oncomorphology of cervical cancer are characterized by the accumulation of new information; various molecular biological research methods aimed at studying the mechanisms of cervical carcinogenesis are being tested and applied; targeted anticancer drugs and biomarkers which are useful for the diagnosis of various stages of the tumor process in cervical cancer are being sought [8,9,10,11,12,13,14,15,16,17,18,19]. The search for reliable genetic, molecular and immunohistochemical markers for early diagnosis of precancerous and neoplastic processes in the cervix is also an important task of current interest in modern oncology. Biomarkers in oncology include certain genes, DNA and RNA molecules, proteins, enzymes, antigens, and other cellular and biological products that can be detected at various stages of carcinogenesis, under the influence of therapy, and are described in many modern reviews and articles concerning carcinogenesis, precancerous lesions, and cervical cancer (Table 1).

**Figure 1:**
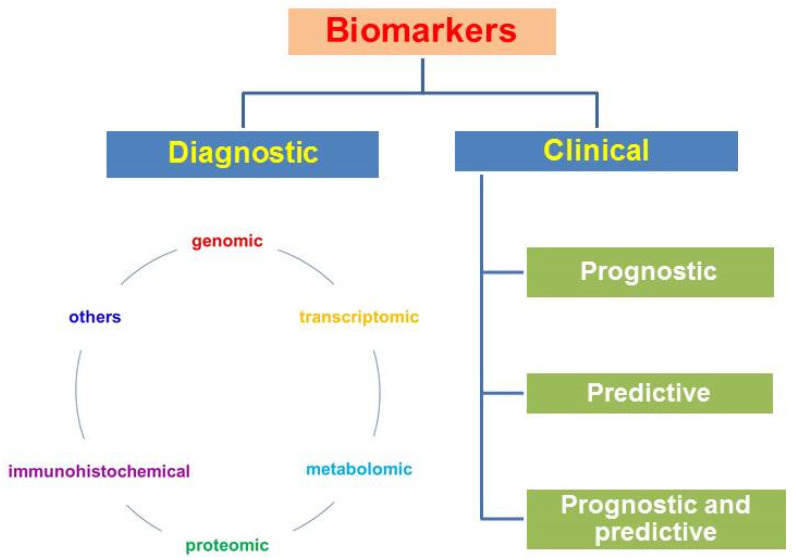
Types of biomarkers for investigation of precancerous lesions and cervical carcinoma.

According to the type of biomolecules and methods of their detection, genomic, transcriptomic, and metabolic factors (immunohistochemical, biochemical and others) are distinguished. Biomarkers can be divided into diagnostic markers and prognostic and predictive clinical factors (Figure 1).

Diagnostic markers make diagnosis possible; clinical biomarkers include the following groups: (1) prognostic ones, which allow one to predict the course of the disease and survival without antitumor treatment; these are factors associated with tumor proliferation, differentiation, angiogenesis, invasion, or metastasis; (2) predictive ones, which allow prediction of the clinical effect, the relapse-free period, survival, sensitivity to treatment, and the toxicity of the planned treatment; they are associated with molecular targets for targeted drugs and a cascade of intracellular signals in a tumor cell; (3) there are factors that can simultaneously have both prognostic and predictive properties [82].

## 2. Etiological Factors of Cervical Cancer

HPV infection has traditionally been considered to be a necessary condition for the development of most types of squamous cell cervical carcinomas. Nevertheless, in recent years, there has been an increase in data indicating that some cervical tumors, mainly adenocarcinomas, are HPV-negative, not associated with HPV infection [1]. The etiological role of HPV oncogenic risk in the development of malignant neoplasms has been proven not only for cervical carcinomas, but also for several other types of cancer—cancers of the vulva, vagina, penis, oropharynx, anal canal, and rectum [11,13,17]. It was established that in squamous cell carcinoma of the head and neck, HPV is found in 25% of patients [13]; HPV type 16 is detected in most cases. HPV has been identified in several cases with breast carcinomas [11]. It was found that carcinomas of the cervix, as well as carcinomas of other localizations, in particular, the pharynx and vulva, not associated with HPV infection, are characterized by a more aggressive course, which has a great clinical importance. Therefore, according to the fifth edition of the WHO classification of tumors of the female genital organs published in 2020 [1], for cervical carcinomas, such categories as HPV-associated and non-HPV-associated cervical tumors are included. For the differential diagnosis of these groups of tumors it is necessary to determine a surrogate marker of HPV infection—p16 or HPV testing [1]. Thirteen types of HPV oncogenic risk have been identified (16, 18, 31, 33, 35, 39, 45, 51, 52, 56, 58, 59, 68). HPVs of high oncogenic risk are of the greatest importance in cervical carcinogenesis; more often in patients with cervical cancer, HPV types 16 and 18 are identified [1,5,6,15].

## 3. Mechanisms of Cervical Carcinogenesis

HPV-associated cervical carcinomas in most cases develop over several years, which allows screening programs to be realized based on the identification of markers of various stages of the tumor process. Several stages characterize the currently accepted scheme of carcinogenesis of HPV infection associated with carcinomas. Integration of human papillomavirus DNA into the genome of cervical epitheliocytes leads to the persistence of viral oncogenes E6 and E7, the main targets of which are p53 and pRb, as well as, according to recent data, other biomolecules, including proteins that affect epigenetic factors and splicing. These changes lead to uncontrolled proliferation and induction of carcinogenesis [1,11,13,15]. In the development of HPV infection, the following clinical periods are distinguished [13]:

1. Latent period: the virus is localized in the keratinocytes of the basal layer of the cervical epithelium; it is not detected by cyto-/histological examination; it is assumed that there is a small number of HPV copies in the episomal state in the basal epithelial cells;
2. Subclinical or productive/replication phase: as a result of HPV reproduction in epithelial cells, the number of its copies increases, protein synthesis and maturation and differentiation of cells are inhibited, and koilocytosis is determined by cyto-/histological examinations; in most cases, the infection is transitional, and the virus is eliminated;
3. Clinical period (facultative precancerous lesion): with manifestation in the form of exophytic condyloma and neoplasia, the virus is persistent; the HPV genome is integrated into the epithelial genome; there is a pronounced violation of the proliferation and differentiation of the epithelium; cyto-/histological changes and signs of intraepithelial neoplastic processes CIN1, HSIL-CIN 2, CIN3, and cancer in situ are revealed.

The main mechanisms of carcinogenesis in HPV-associated tumors of the cervix are largely determined by the structure of the human papillomavirus genome. It is known that the HPV genome is represented by a double-stranded circular DNA with a length of 6.8 to 8 kb and the presence of three separate regions: (1) the early region includes genes (E) that encode molecules for viral replication and regulatory proteins that play a role in different stages of the HPV life cycle: E1 and E2 are responsible for viral replication; E4 takes part in the maturation of virus particles; E5, E6, and E7 are oncogenic; and the key viral oncogenes are E6 and E7; (2) the late area includes genes (L) encoding structural proteins involved in the formation of the virus capsid; (3) the long control area (LCR) contains regulatory elements to control viral DNA replication and transcription [11,15]. With the integration of HPV into the DNA of the epithelial cell, a deletion develops in the site of localization of the E2 gene, which leads to overexpression of the E6 and E7 genes and the overproduction of the E6 and E7 proteins, inducing changes in proteins which are the products of the p53 and Rb genes, and then a violation of the regulation and strengthening of cell proliferation [11]. The E6 and E7 genes play a leading role in carcinogenesis: the products of the E6 and E7 oncogenes cause the stimulation of the ubiquitin-proteasome system (UPS) of the cell, the destruction of the retinoblastoma protein (pRb), and the stimulation of the host cell to the S phase, which provides replication of the virus. At the same time, HPV E6 damages p53, which impairs apoptosis. These intracellular events lead to genomic instability and promote oncogenesis [11].

There is evidence that the integration of the virus into the genome is not observed in all HPV-positive cells. A study of samples obtained from the Cancer Genome Atlas found that the integration of the genome of HPV type 18 took place in 100% of cases, while in samples with HPV type 16, the viral genome was integrated in 76% of cases [16]. In these observations, genetic or epigenetic changes are often found in the regulatory regions E6 and E7, which lead to dysregulation of these two oncogenes. In this way, despite the fact that cancer progression and integration of the HPV genome are separate events, dysregulation of E6 and E7 is the most important event in cervical oncogenesis [11,12,13,14,15,19,82,83]. The main targets of E6 and E7 are p53 and pRb; however, it has been established that there are other factors/targets that determine the oncogenicity of HPV of high oncogenic risk. The least studied are the effects of the E5 oncogene, which can affect the synthesis of host cell DNA [11,84]. It was found that during the integration of HPV into the genome, the expression of E5 is often disrupted; apparently, E5 plays a role at the initial stages of cervical carcinogenesis, and to a lesser extent during its persistence. In addition, E5 appears to contribute to the disruption of keratinocyte differentiation [84].

A complex genomic study of cervical cancer identified new mutated genes and gene amplifications in endometrioid-like cancers, consisting mainly of HPV-negative tumors with a high frequency of the mutations KRAS, ARID1A, and PTEN; it established that in more than 70% of cases of cervical cancer, genomic changes take place in one or both of the PI3K/MAPK and TGFβ signaling pathways [16]. These studies support the existence of various molecular subtypes of cervical cancer [16].

According to different research, in HPV infection and cervical cancer there are various epigenetic changes and DNA methylation processes, and the participation of microRNAs in these processes has been described [18,19,65,83,85,86,87,88]. At present, many of these processes have been studied insufficiently, and their interpretation is ambiguous, so the molecular mechanisms underlying these pathological changes are being investigated [11,14,85]. Recently, Wang et al. (2021) described the epigenetic regulation of the p16 tumor suppressor by miR 29a, which can justify the search for miRNA-based treatment options for cervical cancer [85]. It has been found that during the development of carcinomas, including various stages of cervical cancer, numerous epigenetic changes, such as hyper- and hypomethylation of DNA and tumor suppressor genes and modification of histones associated with the promoter regions of regulatory genes are observed. The results of meta-analysis confirm the hypermethylation of p16INK4a as an epigenetic marker for the progression of carcinogenesis in cervical cancer [65]. Expression of tumor suppressor genes, oncogenes, and growth factors is regulated by many histone-modifying enzymes, such as deacetylases and histone acetyltransferases; changes in the expression of histone modifiers also lead to epigenetic control of gene expression [16]. For example, it was found that DNA methylation is much higher in CIN2 and CIN3 compared to CIN1 [18]; the DNA methylation test is characterized by high specificity and sensitivity as compared to the ASCUS + cytological study and HPV16/18 genotyping [18]. Understanding the role of epigenetic changes in cervical carcinogenesis will help to develop effective methods for the detection and treatment of cervical cancer [8,18,85].

At the moment, the possible role of circular RNAs (circRNAs) in oncogenesis in the development of carcinomas of the organs of the female reproductive system is being investigated. It is believed that in cancer of the ovaries, endometrium, cervix, and breast carcinoma, circRNAs have prognostic, diagnostic, and therapeutic potential [86,87,88,89]. Circular RNAs are a new class of non-coding RNAs. In several types of cancer, abnormal expression of these biomolecules happens, for example, in virus-associated malignant neoplasms—anogenital carcinoma associated with HPV, oropharyngeal and oral cancer [89]. It has been shown that circRNAs are involved in oncogenesis, cancer progression, and drug resistance, and some of them are useful diagnostic and prognostic markers.

In cervical cancer, aberrant expression of miRNA, circRNA, and IncRNA has been revealed, making a new contribution to the understanding of the molecular mechanisms of oncogenesis, and these molecules may turn out to be potential biomarkers for oncological screening and monitoring of cancer recurrence [87,88,89]. Long non-coding RNAs (LncRNAs) (>200 nucleotides) are non-coding transcripts, and some of them have been associated with the development, progression, and metastasis of certain neoplasia, such as leukemia and breast, colon, and liver cancers. LncRNA can play a role at the transcriptional and post-transcriptional levels, contributing to the development of various diseases, drug resistance, signal modulation, DNA repair, the cell cycle, and apoptosis. Patterns of aberrant expression of some LncRNAs have been found in cervical carcinoma cells and in precancerous lesions of the cervix; nevertheless, molecular mechanisms and biological effects associated with LncRNA require further research [90]. The role of long non-coding RNAs associated with HPV and cervical cancer, which can play a role in the processes of cell proliferation, migration, invasion, and apoptosis, is being investigated. LncRNAs can be seen as candidates for prognostic and predictive markers; however, molecular mechanisms involving LncRNAs, ncRNAs, and mRNAs have not been elucidated yet and are subject to further clarification [90]. It is known that approximately 20–40% of high-grade intraepithelial neoplasms regress spontaneously; however, an increase in the 3q chromosomal region, in which the human telomerase RNA gene is localized at 3q26, correlates with the degree of disease in CIN and cervical cancer lesions. In a study of an increase in the 3q26 gene as a genetic marker for the prognosis of neoplasia using in situ hybridization, a connection with the prognosis was established; the absence of an increase in this DNA region during a diagnostic biopsy may indicate a high probability of regression of the disease [20].

## 4. Biomarkers of HPV Infection and Carcinogenesis in Cervical Cancer

Potential diagnostic and prognostic biomarkers in most neoplastic lesions of the cervix are based on molecular mechanisms associated with HPV infection [10,11,13]. Clinically significant markers of HPV-mediated oncogenesis are used in the following methods: determination of virus by PCR methods, assessment of viral load, detection of E6/E7 mRNA, p16INK4a methylation, p53, Rb and p16INK4a, other cell cycle biomarkers, proliferation and apoptosis, determined by immunohistochemistry [11,21,22,27]. The most widely used and well-studied biomarkers are HPV DNA in cervical epithelial cells and the immunohistochemically detectable expression of the p16INK4a protein, which is directly related to HPV oncogenic risk and Ki-67, which is a proliferation marker [10,11,12]. The p16 protein takes part in the regulation of the cell cycle and is an inhibitor of the activity of the cyclin-dependent kinase CDK4/6; normally its concentration in the cell is extremely low. Overexpression of p16 indicates active expression of the HPV E7 viral oncogene and inactivation of the retinoblastoma gene by the E7 virus protein; this is observed in malignant neoplasms caused by HPV of high oncogenic risk [10,11,12]. From 10% to 25% of cervical carcinomas are adenocarcinomas of various histotypes; high-risk HPV DNA is found in 94% of lesions in situ, 85% of adenosquamous carcinomas, and 76% of adenocarcinomas [23]. HPV DNA is most often found in endocervical adenocarcinoma of the usual type (90%), less often in serous (30%), clear cell (27%) and endometrioid carcinoma (13%). A surrogate marker of HPV is overexpression of p16, detected immunohistochemically; p16 is a sensitive and specific marker not only in the diagnosis of squamous cell neoplasms and squamous cell carcinoma, but also in identifying several histotypes of glandular neoplasms of the cervix [23].

HPV infection can lead to aneuploidy, chromosomal aberrations, and DNA hypermethylation. Potential biomarkers in cervical neoplasia are methylation factors and chromosomal abnormalities in cervical carcinoma detected by fluorescence in situ hybridization (FISH), in particular in the region of the telomerase RNA gene (TERC) located in the 3q26 region [20]. It has been established that E6 proteins of oncogenic HPV promote transcription of telomerase reverse transcriptase (TERT), which restores DNA at the telomeric end of chromosomes and therefore increases the cell division limit [11]. Increases of chromosome 3q, which contains the telomerase sequence (TERC), and chromosome 5p, which contains the TERT gene, are associated with intraepithelial neoplasia, which is a useful marker for detecting progressive lesions [8,11,15]. Telomerase is an enzyme which with an increase of its activity, compensates for the shortening of telomeres during sequential replications, which is observed during cell division; an increase in telomerase activity is characteristic of stem and tumor cells, and telomerase activity is not detected in most somatic cells [15]. It was found that the telomerase RNA gene in the 3q26 region is found in CIN lesions and cervical carcinoma and correlates with the degree of the disease. It was demonstrated by fluorescent in situ hybridization that patients without an increase in 3q26 showed regression of the disease, i.e., the lack of 3q26 increase on diagnostic biopsy can potentially be used to identify high-grade CIN lesions with a high probability of disease regression, preferably as part of a wider biomarker panel [20]. The authors suppose that 3q26 FISH may help select women with low-grade cervical lesions (LSIL) who do not need immediate colposcopy and patients with severe lesions (HSIL) who do not need immediate treatment. Such approach can help to reduce treatment costs and reduce the side effects of surgery treatment; however, further studies on larger sample groups are needed to confirm this hypothesis [20].

Serum biomarkers play an important role in the monitoring of many malignant neoplasms, including cervical cancer [24,25]. A well-known biomarker is the squamous cell carcinoma antigen SCC-Ag, which was discovered in 1977. The squamous cell carcinoma antigen (SCCa) is the most widely used and most reliable tumor marker for squamous cell carcinomas [24]. During the analysis of the literature devoted to the study of the level of SCCa in the blood serum as a predictor of cervical cancer metastases to lymph nodes, it was found that SCCa can serve as a reliable prognostic factor; the meta-analysis confirmed the value of the predicting property of SCCa, even though the predictive value was moderate. Elevated SCCa is clinically relevant information in addition to CT scan or magnetic resonance imaging (MRI) supporting the need for laparoscopic evaluation of the pelvic and/or para-aortic lymph nodes [24]. In a meta-analysis of the association of serum SCC-Ag with relapse and mortality in patients with squamous cell carcinoma of the cervix, it was found (in 61 articles) that SCC-Ag in serum was invariably associated with relapse and mortality in newly diagnosed cervical cancer; this marker may be useful for monitoring disease progression in patients with cervical cancer [25].

Despite the huge progress that has been made in the screening and management of women with HPV-associated cervical pathology, there is still a need for clinically reliable biomarkers to further improve the screening, diagnosis, and prognosis of cervical carcinomas [8,10,21,22]. An important role in clinical pathology and oncologic morphology is given to immunohistochemical methods aimed at solving diagnostic problems and searching for new prognostic and predictive factors. Taking into account the role of individual biomolecules in the cell during the development of physiological and pathological processes, the following categories of immunohistochemical biomarkers are distinguished –markers of the cell cycle, proliferation, apoptosis, adhesion, invasion, angiogenesis, microenvironment, immune factors, and others—which are currently being tested for verification of “cancer stem cells” [56].

## 5. Markers of Cell Cycle and Proliferation

The main biomarkers associated with the cell cycle, proliferation, and apoptosis in cervical neoplasms include Ki-67, cyclin-dependent kinases and their inhibitors, surrogate marker of HPV infection p16INK4a which is associated with active expression of HPV E7), pRb protein, p53 gene products, anti-apoptotic protein BCL-2, and pro-apoptotic protein BAX.

The most commonly used immunohistochemical markers for verifying the phases of the cell cycle [27] are the following: (1) Ki-67, which detects proliferating cells in different phases of the cycle, appears at the end of the G1 phase, increases in the S phase, and is maximally expressed in the mitotic phase; PCNA-proliferating cell nuclear antigen; (2) cyclin-dependent kinases (Cdk), which are activated by subunits: cyclins D (D1-D3) are involved in the transition of the cell from the G0 phase to G1; cyclin E, from G1 to S; and cyclin B, entering the mitosis phase; in practice, cyclin D1 is widely used; (3) inhibitors of cyclin-dependent kinases (CKI), which block the activation of various kinases; some of them can inhibit several Cdk (p21CIP4, p27KIP1, p57KIP2); others, individual complexes of cyclins with the corresponding Cdk-complexes.

For the verification of neoplastic processes (squamous cell carcinomas, adenocarcinomas), as well as for the differential diagnosis of various stages of HPV-associated carcinomas and metaplastic processes, Ki-67, Cyclin D1, and p16INK4a are most often used, being associated with the expression of HPV E7 during infection with oncogenic types of the virus, and with the anti-apoptotic p53 gene product. In several neoplasms, autonomous cell proliferation is accompanied by overexpression of cyclin D1 due to amplification of the 11q13 chromosome gene, the absence or presence of an inactive form of the pRb protein in the cell nucleus, and increased expression of MCM2 (mini chromosome maintenance protein 2) [27].

In a significant number of publications in the field of precancerous lesions and cervical cancer, the expression of the proliferation marker Ki-67, often in combination with other antigens, has been studied using immunohistochemistry or immunocytochemistry [12,28,29,30,31,32]. In a meta-analysis to assess the relationship between Ki-67/MIB-1 and cervical cancer (13 studies, 894 patients), it was found that this marker can be used as a predictor of prognosis due to its high correlation with the prognosis of the disease [28]. A meta-analysis (7 articles, 2628 patients) assessing the accuracy of double immunocytochemical staining of p16/Ki-67 testing for high-risk HPV demonstrated that this diagnostic test is characterized by high sensitivity and moderate specificity in the study of PAP smears with abnormalities for HSIL and cervical carcinoma [29]. A combined assessment of the expression of Ki-67 and p16ink4a biomarkers in combination with H&E staining was used to objectify the differential diagnosis of CIN1-CIN3 intraepithelial neoplasms, and E4 and hypermethylation in the epithelial cells of the cervix were proposed as potential biomarkers of cervical cancer [30]. In the examination of 58 patients with intraepithelial neoplasia of the cervix of various degrees, three immunohistochemical markers were used—Ki-67, p53, and p63. It has been found that there is a significant increase in the expression of biomarkers in people with CIN III compared to CIN II and CIN I; besides this, in CIN I the expression of p53 and p63 was significantly more pronounced in comparison to the control. The authors believe that the markers p53 and p63 are reliable and can distinguish reactive changes from CIN I, while all three biomarkers (Ki-67, p53, and p63) showed a high degree of sensitivity and specificity for the differential diagnosis of CIN III, CIN II, and CIN I [31]. Numerous publications indicate that markers such as Ki-67 can be considered useful for the differential diagnosis of the stages of cervical neoplasia progression [30,31,32]. Thus, a review study conducted on the basis of analysis of 28 publications and devoted to the assessment of the immunohistochemical expression of Ki-67, p16, and p53 in precancerous lesions and cervical cancer indicates that high expression, especially of p16 and Ki-67, is observed in more severe lesions, while in normal cervical epithelium it is minimal or absent [32].

Ki-67/MIB1 is considered to be a prognostic marker of cervical cancer [29]; however, other features of the disease, such as the degree of malignancy, damage to the lymph nodes, and metastasis, may influence patient survival [28,33]. It should be noted that despite the fact that it is well-known that Ki-67/MIB-1 is present in the nuclei of cells in the G1, S, G2, and mitotic phases, nevertheless, the mechanism of expression of the Ki-67 gene is still unknown [33], and for an accurate quantitative assessment of the degree of expression of the Ki-67 marker, a comparative analysis of the results obtained in various studies, unified methods for calculating expression indicators are required, possibly with the use of digital microscopy methods [30,34].

A meta-analysis of studies of double immunocytochemical staining of cytological smears with p16/Ki-67 demonstrated that this test is characterized by a high level of sensitivity and moderate specificity for detecting squamous cell intraepithelial lesions and cervical cancer, i.e., p16/Ki-67 double staining may be a reliable adjunct method for detecting high-grade squamous intraepithelial lesions (HSIL) in pathology in women by PAP testing. However, no meta-analysis studies have tested the accuracy of p16/Ki-67 double staining for the interpretation in the cases of glandular neoplasms of the cervix [29]. It has been established that TOP2A and Ki-67 antibodies can be used to screen for cervical cancer in immunocytochemical examination of cervicovaginal smears; in cervical cancer, there is an increased expression of nuclear proteins Ki-67 and type IIa DNA topoisomerase (TOP2A) [35]. During an evaluation of the immunohistochemical expression of Ki-67 protein in combination with p16, cytokeratin 7 (CK7), and histomorphometry in cases of cervical LSIL, it was established that these markers may be useful for identifying LSIL with a tendency toward progression of the neoplastic process. Thus, p16, CK7, and Ki-67 may be useful biomarkers for identifying LSIL lesions of the cervix that require special attention [36].

Expression of cyclin D1 in CIN and cervical cancer was evaluated in comprehensive research on the relationship between the levels of immunohistochemical expression of protein and mRNA of ovarian cancer gene 1 (OVCA1), cyclin D1, and p16 and infection with high-risk human papillomavirus [26]. It was found that the expression of proteins OVCA1, cyclin D1, and p16 increased in CIN and cervical cancer. Significant differences were revealed in the levels of expression of OVCA1 and p16 compared to the norm in cervical cancer tissues and areas with CIN, between CIN and carcinoma tissues, and in the expression of the cyclin D1 protein in normal tissue and in the area of cervical cancer, and in the areas of CIN and cervical cancer, while not there was a significant difference in cyclin D1 expression levels between normal and CIN tissue. The positive abnormal expression of the OVCA1 protein and its synergistic effect with various genes (oncogenes, tumor suppressor genes, and other regulatory factors) associated with cancer were revealed; this fact indicates its possible role in the occurrence and development of cervical carcinomas. The role of OCVA1 in cervical cancer has been investigated in a small number of works; therefore, the mechanisms by which OVCA1 participates in realizing cellular functions and interacts with associated genes or factors, including p16 and HPV, are not clear, but, according to the authors’ opinion, OCVA1 may be a potential biomarker for diagnosis cervical carcinomas [26].

## 6. Markers of Apoptosis

The products of the p53 gene, the anti-apoptotic protein BCL-2, and the pro-apoptotic protein BAX play an important role in the processes of apoptosis in normal and tumor cells. The p53 gene is associated with the regulation of several cyclin-dependent kinases, stopping the cell cycle after DNA damage to restore the latter before replication and cell division, which provides genome stability. If repair is not possible, p53 stimulates the transcription of pro-apoptotic genes such as bax and fas, i.e., it induces cell apoptosis, which helps to prevent the occurrence of mutations and neoplastic cells. P53 affects the expression of various proteins involved in the induction of apoptosis, regulation of the cell cycle, inhibition of growth and angiogenesis, and possibly on other intracellular processes [27]. Normally, p53 is inactivated rapidly, while in many tumor cells under conditions of genetic instability, in the presence of mutations in the p53 gene, the expression of the p53 protein increases, which is a marker of the development of the neoplastic process. In many malignant neoplasms, including cervical cancer, endometrioid endometrial cancer, and serous ovarian carcinoma, an increase of expression or aberrant expression products of the p53 gene localized on chromosome 18 is observed [10,27].

The regulators of apoptosis also include the anti-apoptotic protein Bcl-2 (B cell lymphoma/leukemia-2) and the pro-apoptotic protein BAX, which affect the function of cell caspases; in many neoplasms, an increase in Bcl-2 expression is often observed. Bcl-2 is one of the main inhibitors of apoptosis, localized mainly on the external membrane of mitochondria and comprising 16 members. Some of them inhibit apoptosis (Bcl-2, Bcl-XL, etc.), while others activate it (Bax, Bad, Bid, etc.) [37]. BCL2, BCL-XL, and BAX affect mitochondrial morphology and cell metabolism regardless of the mechanisms of cell death; BCL2 and BCL-XL inhibit apoptosis and are important anti-apoptotic proteins which integrate into intracellular membranes, including the endoplasmic reticulum, and inhibit apoptosis by binding pro-apoptotic proteins of the BCL2 family, such as BAD and BAX, with the formation of inactive complexes [38]. Bcl-2 is present in many normal cells; in the mitochondria, it stabilizes the membrane and prevents the release of several factors that induce apoptosis. When the Bcl-2/Bax complex is formed, the former loses its inhibitory activity. Before receiving the signal for apoptosis, Bax is in the cytoplasm, and after the signal it migrates into the mitochondrial membranes, makes them permeable, and stimulates the release of cytochrome C and AIF (apoptosis-inducing factor) from the membrane space into the cytosol. The latter penetrates into the nucleus, where it causes chromatin condensation and fragmentation of the nucleus, followed by activation of apoptotic caspases: cytochrome C activates factor Apaf-1, which stimulates procaspase 9, which activates the caspase cascade with subsequent cytological changes which are typical of apoptosis [37].

Apoptosis plays an important role in the radiation sensitivity of cancer cells. Proteins of the BCL2 family, such as BCL2, BCL-XL, and BAX, are among the most important radiation-related regulators of apoptosis [38]. There are publications indicating that in cervical cancer, the immunohistochemical expression of BCL2 and BAX is associated with the progression of the disease and the radiosensitivity of tumor cells. Thus, when assessing the prognosis of patients with primary cervical cancer and recurrent carcinoma, a positive staining for BCL2 was associated with a better 5-year survival rate. In a study of radioresistant and radiosensitive cervical cancer, it was found that after irradiation, patients with BAX-positive tumors showed a significantly better response compared to patients with BAX-negative tumors, whereas patients with BCL2-positive tumors showed a significantly weaker response compared to patients with BCL2-negative tumors; increased BAX expression after radiation therapy correlated with good survival, while increased BCL2 expression correlated with poor survival. Expression levels of BAX and BCL2 after radiation therapy are useful prognostic markers in patients with human cervical carcinoma. Thus, in cervical cancer, expression of BCL2 and BAX, as well as p53, is associated with the tumor’s response to radiation therapy [38,39]. Chemotherapy and radiation therapy probably cause epigenetic changes in the pattern of DNA methylation and changes in the levels of transcripts of several genes in the tissues of invasive cervical cancer—after treatment methylation of the promoters of the BAX, BRCA1, and ESR1 genes increased significantly, while the expression of transcripts of the MYOD1 and MLH1 genes was significantly reduced [40]. These facts may have significance for understanding the biology of the tumor’s response to radiation therapy.

When studying the relationship between sensitivity to nedaplatin (NDP) and immunohistochemical expression of such biological factors in cervical cancer as Ki-67, p53, Bcl-2, Bax, cleaved caspase-3, and COX-2, it was found that low or negative expression of p53, Bcl-2, and COX-2 and high or positive expression of cleaved caspase-3 were significantly correlated with high sensitivity to NDP, whereas there were no significant differences in Ki-67, Bax, or ERCC1 expression between the low and high sensitivity groups. According to data, susceptibility to platinum can be predicted by immunostaining for these specific factors in biopsies or surgical specimens before treatment, and expression profiles of these markers may provide additional information for planning individual chemotherapy for cervical cancer. Thus, individualized chemotherapy to maximize the effectiveness of treatment may become clinically valuable in the cases of cervical cancer [41]. Negative correlation of the expression profile of the anti-apoptotic proteins SAG, Bcl-xL, and p53 with the overall survival of patients with cervical cancer stages IIB-IVA has been demonstrated. The expression profile of the anti-apoptotic protein SAG, mitochondrial apoptotic proteins Bcl-xL and Bak, and tumor suppressor proteins p73 and p53 was studied by real-time PCR to evaluate their relationship with clinical parameters and survival during the observation period [42]. No association of proteins of the apoptotic pathway with the clinical and pathological characteristics of patients was found; no significant differences in the expression of proteins SAG, Bcl-xL, Bak, p73, and p53, depending on the stage and degree of the tumor, were revealed. Low expression levels of SAG, Bcl-xL, and p53 were found to be useful as prognostic predictors in patients with cervical carcinoma [42].

It is known that 80–95% of cervical cancer cases are caused by HPV infection; among them the main part are squamous cell carcinomas (SCC), but currently the quantity of cervical adenocarcinomas is increasing, for the treatment of which combined radiation and chemotherapy is used [43,44]. Numerous biomarkers have been investigated in CIN and cervical cancer; the correlation between HPV infection and overexpression of commonly used biomarkers, such as p53 and bcl2, has not always been established in all cases, as there is no significant relationship between HPV 16/18 infection and p53 and bcl2 expression in precancerous and malignant lesions. However, differences were found between bcl2 expression in the cases with CIN compared with cervical carcinomas [44]. Most likely, further studies are needed to assess the relationship between HPV infection and overexpression of p53 and bcl2 proteins, and while Bcl2 expression can be used as a diagnostic marker for differentiating malignant tumors from precancerous lesions, its role as a prognostic marker also needs further evaluation [44]. The mechanisms of resistance to therapy of various types of cervical cancer are complex and insufficiently studied; various panels of biomarkers associated with several biological functions, such as apoptosis, cell adhesion, DNA repair, hypoxia, metabolism, pluripotency, and proliferation, are being investigated [43]. The updated WHO 2020 classification indicates the importance of differences between HPV-positive and HPV-negative tumors—there is evidence that cervical adenocarcinomas have molecular peculiarities both between different types within the group and in comparison with squamous cell carcinomas. ERBB2 (HER2) mutations and PD-L1 expression are considered to be promising prognostic markers in cervical adenocarcinomas, but at present this question is not completely clear [43].

## 7. Expression of Cytokeratins—Markers of Epithelial Differentiation

Expression of various cytokeratins in the epithelium is typical of both normal cellular elements and malignant neoplasms of epithelial genesis of the cervix [27,36,37,45,46,47,48]. In malignancy of the cervical epithelium, there is a violation of the cytokeratin profile of the epithelial layer; changes in the expression of various cytokeratins—CK7, CK8, CK17, and CK19—are described [37,48]. Cytokeratins (CK) are intermediate filaments of the cytoskeleton, which are markers of epithelial differentiation; 20 subtypes of CK have been described, identified in different types of human epithelial cells [37,48,49]. Normally, the expression of CK7 and CK19 is found in the monolayer epithelium of the gastrointestinal tract, bile ducts, pancreas, pulmonary alveoli, endometrium, and collecting ducts of the kidneys; in transitional epithelium cells, CK19 can be expressed in the basal layer of stratified squamous epithelium (except for the skin); CK17 is normally found in the basal layers of the epithelium [37]. CK8 is expressed in reserve cells of the cervix, epithelial cells of the glands, and immature squamous metaplasia, but not in squamous epithelial cells. CK17 is found in reserve cells and immature metaplastic cells, but not in squamous epithelium or areas of mature squamous metaplasia. The CK isotype depends on the type of cells and their localization. It has been described that CaM-kinase-like 1 (DCAMKL-1), Lgr5, CD133, α-fetoprotein, cytokeratin-9 (CK19), Lin28, and c-Myc are activated in hepatocellular carcinoma cancer stem cells (CSCs); DCAMKL-1 may be a specific marker of CSCs [48].

Epithelial cells can change the cytokeratin profile in response to various damages; during regeneration and the metaplastic process, defective variants of cytokeratin expression have been described [37]. CK8 and CK17 are subtypes found in the tissues of CIN and cervical cancer [48]. There are publications that have described the immunohistochemical expression of cytokeratin CK7 and CK19 in HPV-associated carcinomas of the cervix [36,45,46,47]. It is known that CK8, CK18, CK19, and often CK7 express adenocarcinomas [27]. In an analysis of the expression of CK7, CK19, and p16 in HPV-associated cervical carcinomas, it was found that both CK7 and CK19 are expressed in cervical neoplasms [45]. Cytokeratin CK7 is a marker of the cervical squamous junction, in the area of which as is traditionally believed, infection of HPV epithelial cells of high oncogenic risk is observed. Despite the possible link between CK7/CK19 and cervical cancer, the mechanisms of CK7/CK19 involvement in HPV-mediated cervical carcinogenesis remain poorly understood [45,48]. When studying the expression of CK7, CK19, and p16 in 25 cases of high-grade intraepithelial neoplasia of the cervix (CIN3) and in 30 cases of squamous cell carcinoma, Lee at al. found that the expression of CK19, p16, and HPV was positive in all cases of CIN3 and squamous cell cervical cancer, while the expression CK7 was positive in all cases of CIN3 and in 66% of cases of squamous cell carcinoma. The authors assumed that CK7 may be more associated with viral episomal replication, and CK19, with viral integration, which promotes viral replication and malignant transformation in HPV-infected cells [45]. Da Costa et al. (2017) presented results suggesting the possibility of using immunohistochemical staining using antibodies to CK7, p16 protein, and Ki-67 to detect LSIL lesions of the cervix with a tendency toward progression of the neoplastic process [36]. It was shown that CK8 and CK17 are of great importance in the diagnosis of CIN and cervical cancer. In cervical cancer cells, overexpression of CK17 takes place; moreover, in tumors with a higher expression of CK17, a more pronounced tendency toward metastasis is observed. Recent studies emphasize the importance of CK19 in cervical cancer stem cell verification; however, the function of CK19 in cervical cancer stem cells is still unclear [48].

## 8. Markers of Cell Adhesion, Invasion, and Metastasis

Moreover the processes of proliferation, apoptosis, and cell differentiation, intercellular interactions and factors of adhesion, invasion, and metastasis—such as E-cadherin, CD44, and matrix metalloproteinases—are of great importance in the carcinogenesis of epithelial tumors. E-cadherin (E-cad), a transmembrane glycoprotein, provides the strength of intercellular connections of epithelial cells and is involved in the control of migration, growth, and differentiation. E-cad has an extracellular, transmembrane, and intracellular component; normally, the extracellular regions of the molecule of epithelial cells form strong connections and the cytoplasmic components interact with cytoplasmic proteins’ catenins (beta and gamma catenin), which bind with alpha-catenin, which provides connection of the cadherin-catenin complex with the actin cytoskeleton. It is supposed that E-cadherin is involved in the regulation of p53 activity, i.e., it can affect the processes of the cell cycle and apoptosis [27]. Aberrant expression of cadherins has been demonstrated during invasion and metastasis of tumors; their participation in carcinogenesis and progression of cervical cancer has not yet been studied sufficiently. It was found that there is a gradual decrease in E-cad expression and an increase in P-cad during the development from CIN to cervical squamous cell carcinoma, the expression of cadherins or E-P switching was associated with some clinical parameters indicating poor prognosis and patient survival, and immunohistochemical staining to detect E-cad and P-cad is useful in the diagnosis of CIN and the prognosis of disease at an early stage of cervical cancer [50].

It has been established that ADAM9 protease (ADAM family) takes part in the processes of cell adhesion, migration, and signaling; overexpression of ADAM9 has been described in many solid tumors, such as cancer of the prostate, kidney, pancreas, lungs, and stomach. Disintegrin-metalloproteinases ADAM9, ADAM12, and ADAM15 are activated in gastric cancer [51]. Immunohistochemical evaluation of the expression of ADAM9 protease in normal epithelium, CIN3 lesions, and squamous cell carcinoma of the cervix (50 cases) showed that in the normal epithelium of the cervix, there was weak cytoplasmic expression and membrane staining of ADAM9; pronounced expression of ADAM9 was observed in the foci of CIN3, and to a greater extent in squamous cell carcinomas of the cervix. Thus, it was established that the expression of ADAM9 is low in the squamous epithelium of the cervix and increases in the foci of CIN3 and the tissue of squamous cervical cancer; to identify the diagnostic and prognostic value of ADAM9 in cervical cancer, further studies are recommended [51]. The reported results obtained by Zubel et al. (2009), according to which the ADAM9 protein was found to be expressed in 93% of cervical cancer cases, generally correspond to the data of Isa et al. (2019), who identified ADAM9 in a slightly smaller number of carcinomas—75.8%—which may be due to methodological differences [52]. The authors suppose that the prevalence of ADAM9 expression is high in aggressive tumor types and, taking into account the detected ADAM9 expression in various cervical cancer histotypes, they recommend further studies of the relationship between ADAM9 expression and tumor development [52].

Invasion and metastasis of tumor cells are associated with the processes of destruction of the extracellular matrix, in which matrix metalloproteinases and their inhibitors are involved. Matrix metalloproteinases (MMPs) are a family of enzymes (about 25) capable of destroying the extracellular matrix and basement membrane and performing several important regulatory functions; normally, there is a small amount of MMPs in tissues, the main MMPs are secreted enzymes, and six MMPs are membrane-bound MMP–MT-MMP [53,54]. MMP activation leads to proteolysis and destruction of the intercellular substance, which promotes invasion and metastasis. Normally this process is controlled by tissue inhibitors of metalloproteinases (TIMP), such as TIMP-1, TIMP-2, TIMP-3, and TIMP-4; in many tumors, there is a local increase in the level of MPP [27,53]. Tissue collagenases (MMP-1, MMP-8, MMP-13, MMP-14) and gelatinase (MMP-2, MMP-9) play an important role in the processes of invasion and metastasis [54]. Collagenases hydrolyze fibrillar collagens of types I, II, III, V, and IX; the latter are resistant to the action of proteolytic enzymes, and the products of their hydrolysis are capable of being exposed to a wide range of proteinases. Thus, MMPs provide the development of destructive processes in the interstitial tissue, gelatinases are involved in collagen hydrolysis Type IV, which is the basis of the basement membranes [54]. Tissue MMP inhibitors are characterized by selective specificity; however, they can inhibit the activity of all members of the MMP families. In the blood, a2-macroglobulin is the main MMP inhibitor. The main activators of pro-MMPs include serine proteases: plasmin (for secreted MMPs) and furin (for membrane-bound MMPs, which include MMP-14), which activates pro-MMPs in the Golgi complex [54].

In an investigation of tissue samples of squamous cervical cancer, it was revealed that there is an increase in the expression of MT1-MMP—interstitial collagenase associated with the membrane, and an endogenous activator of its activity—furin. It was found a low expression of MT1-MMP tissue inhibitor TIMP-2, which makes the main contribution to the destructive (invasive) potential of the tumor; expression of MT1-MMP was detected in morphologically normal tissue adjacent to the tumor [53]. Expression of MMPs and their endogenous regulators in cervical cancer tumor samples associated with HPV16 showed that the invasive and metastatic potential of the tumor is apparently determined by an increase in the expression of collagenases MMP-1, MT1-MMP, and gelatinase [54].

In a review dedicated to the analysis of the results of the authors’ own long-term studies, Solovieva et al. described the features of the expression of matrix metalloproteinases and their endogenous regulators in fibroblasts transformed by oncogene E7 HPV-16 (TF), in immortalized fibroblasts (IF), in cell lines associated with HPV-16 and HPV-18, and in clinical samples of tumor tissues of squamous cell carcinoma of the cervix associated with HPV-16 [54]. The results of a study of cervical squamous cell carcinoma tissues indicate that the main contribution to the invasive and metastatic potential is made by an increase in the expression of collagenases—MMP-1 and MMP-14 and gelatinase–MMP-9 as well as a decrease in the expression of inhibitors—TIMP-1 and TIMP-2 and to a less extent, increases in the expression of MMP-2, MMP-1, and MMP-9 can serve as markers for the development of invasive and metastatic potential in squamous cell carcinoma of the cervix. Moreover, in the morphologically normal tissue adjacent to the tumor, significant expression of MMP-1, MMP-2, and MMP-9 was found, which additionally contributes to an increase in the destructive potential of the tumor [54].

In research on tissue of uterus obtained during hysterectomy in patients with cervical squamous cell carcinoma, the expression of membrane-bound matrix metalloproteinase MT1-MMP (MMP-14), its tissue inhibitor TIMP-2, and its activator proMMP-14 furin was investigated [55]. It was established that in cervical cancer tissue, the immunohistochemical expression of MMP-14 was greatly increased, while in normal tissues of the endometrium and myometrium, the expression of MMP-14 was absent or insignificant. However, MMP-14 mRNA has also been found in normal uterine tissues. Furin activity in the tumor was significantly higher than in normal tissues. TIMP-2 expression was low or absent in both tumor and normal tissues. The expression of TIMP-2 mRNA was quite pronounced in both the tumor and normal tissues. The authors demonstrated that the expression of MMP-14 and regulators of its activity in squamous cell carcinoma of the cervix aim to increase the invasive potential of the tumor in the pericellular space and can occur in morphologically normal uterine tissues, which is important for understanding the mechanisms of various stages of cervical carcinogenesis [55].

## 9. Biomarkers of Cancer Stem Cells in Cervical Carcinomas

In recent years, the concepts of tumor (cancer) stem cells (CSCs) and tumor microenvironments (niches) have been suggested and developed [57,91]; various biomarkers of tumor stem cells, including immunohistochemical ones, are being investigated. According to the modern concept, the CSC population is a small fraction of the total quantity of tumor cells, characterized by the expression of certain surface markers that allow it to be identified and isolated, support the growth of a heterogeneous population of tumor cells, form a separate pool of cells, be verified by biological and physicochemical methods, and have an unlimited capability of self-renewal and differentiation in various directions with the formation of a heterogeneous population of tumor cells consisting of different clones, and it is characterized by high resistance to therapy [57]. Resistance to therapy may occur due to the selective expression of some members of the multidrug resistance transporter family, an increase in the expression of anti-apoptotic molecules, an increased ability to repair DNA, or activation of stem cell-specific survival signals (pro-survival signaling), namely Notch, Hedgehog (Hh), Wnt, JAK/STAT, and others [57].

An important role in the structure of the tumor is assigned to the CSC microenvironment, the so-called “niches”. Niches are certain anatomical compartments in tissue surrounding stem cells which regulate the participation of these cells in the formation, maintenance, and repair of tissue [48,57]. The microenvironment includes such structures and components as the extracellular matrix, mesenchymal and endothelial cells, cells of the immune system, adhesion molecules, growth factors, cytokines and their receptors, and blood vessels [57]. The tumor microenvironment is represented by a stroma with cells of various types: fibroblasts, tumor-associated fibroblasts, myofibroblasts, smooth muscle cells, endotheliocytes, pericytes, neutrophilic and eosinophilic leukocytes, basophils, mast cells, T- and B-lymphocytes, macrophages, and dysplasia [91]. According to Zibirov and Mozerov (2018), understanding the mechanisms of interaction between tumor cells and the microenvironment is promising for increasing the effectiveness of treatment [49].

Currently, there is a significant number of publications dedicated to different problems associated with CSCs of various tumors. The tasks of verifying CSCs by detecting cell markers (antigens) by immunohistochemical methods are urgent. A large number of experimental works and reviews are devoted to the identification of surface antigens characteristic of CSCs in various tumors, including cervical cancer, and the list of such markers is constantly growing [48,56,58,59,60,61,62,63,64]. Recently, it is believed that cervical cancer develops from stem cells of the transformation zone upon infection with HPV of high oncogenic risk, where the transformation zone is a niche for cells with a unique expression profile and embryonic characteristics [48]. Modern data on CSCs in cervical cancer are, however, incomplete. It has been established that one of the markers of cervical CSC is Nanog, a factor that is expressed mainly in the internal cell mass of blastocysts; its expression is significantly expressed in early embryonic stem cells and decreased in the processes of differentiation. Nanog plays an important role in the regulation of pluripotent cells in embryogenesis, and recently it was found to be closely associated with oncogenesis; it is found in cancer of the stomach and breast, glioblastoma, lung cancer, and various human sarcomas [48,58]. There is evidence of a pronounced expression of Nanog in the cervix with CIN-II and -III and in cervical carcinoma cells, in contrast to weak expression in CIN I and in normal epithelium [58]. Ye et al. (2008), in an immunohistochemical study of the cervix of 235 patients, showed that the expression of Nanog, nucleostemin (NS) and musashi1 (Msi1) was significantly higher in cervical cancer compared to CIN, and in CIN compared to normal cervical epithelium; the results give evidence that Nanog, NS, and Msi1 may be involved in the carcinogenesis and progression of cervical cancer [58]. Recent studies have demonstrated the expression of CK 17 and CK19 on CSCs; however, further research is required to understand the possible role of CK19 in CSCs, as the study of cervical CSCs is currently at an early stage, so there are no internationally recognized markers of the cells [48].

An increase in the expression of markers such as NANOG, SOX2, and KLF4, as well as CD133, Cd44, ALDH1, CK17, p63, CK8, NS, MSI1, CD49f, ABCG2, BMI1, PIWIL2, and LGR5, was detected in cervical CSCs [56]. In neoplastic processes of the cervix, ALDH1A1 and OCT4 are considered to be candidates for the role of immunohistochemical markers of cancer stem cells for early detection of tumors and monitoring during treatment [59]. The ALDH family includes 19 different isoforms localized in the cytoplasm, mitochondria, or nucleus and involved in the oxidation of intracellular aldehydes, which leads to resistance to several alkylating agents in cancer therapy. ALDH1A1, ALDH1A2, ALDH1A3, and ALDH8A1 are involved in the oxidation of retinol to retinoic acid in the cytoplasm; they move to the nucleus and initiate the transcription of genes involved in early stem cell differentiation. In addition, ALDH1A1 appears to contribute significantly to most of the ALDH activity in CSCs. There is not much information about the regulation of ALDH1A1 expression in cervical lesions, but the function of ALDH1A1 in squamous cervical cancer has been described in several studies. Thus, CSCs of cervical carcinoma cell lines with high ALDH or ALDH1 activity demonstrate enhanced self-renewal properties, high tumorigenicity, and resistance to treatment. It was found that the transcription and expression of the ALDH1A1 and OCT4 proteins were significantly higher in the cervix of patients with CIN II-CIN III and squamous cell carcinoma of the cervix compared with normal controls; these proteins can be biomarkers in cervical cancer [59]. OCT4 is a known stem cell factor that interacts with SOX2 to maintain the self-renewal and pluripotency of human and mouse embryonic stem cells, and loss or suppression of OCT4 expression is associated with stem cell differentiation. OCT4 is detected in CSCs, which indicates its function in tumorigenesis, metastasis, and resistance to anticancer therapy. In cervical cancer, a combined determination of the markers ALDH, OCT4, and NANOG is recommended; the activity of ALDHA1 and OCT4 is increased in the tissues of cervical cancer and precancerous lesions, as well as in the blood plasma [59]. There is evidence that CSCs are resistant to radiation therapy and chemotherapy; it was established that low expression of p16INK4A and high expression of SOX2 and ALDH1A1 correlated with a poor prognosis in patients with radiation therapy [60]. In a study of such potential cervical cancer biomarkers as Musashi, ALDH1, Sox2, and CD49f using PCR and immunohistochemical methods, it was found that according to the results of PCR, the expression of all markers increased compared to normal cervical tissues, and these CSC markers were associated with a poor prognosis of patients with squamous cell carcinoma of the cervix [61].

There is evidence that Sox2-positive SiHa and C33A cells, as compared to Sox2-negative cells, demonstrate more pronounced abilities for self-renewal, differentiation, and tumor formation [62]. In addition, Liu et al. (2014) found that Sox2-positive SiHa and C33A cells expressed higher levels of several genes associated with SCSs, i.e., cells expressing endogenous Sox2 are CSCs in cervical carcinomas [62]. In a review publication devoted to CSCs in cervical cancer, the following factors are included in the group of cancer stem cell markers: ABCG2, ALDH1, CD133, CD49f, OCT4, OPN, and SOX2 [63]. Nevertheless, at present, many issues related to cervical CSCs have not been sufficiently studied, so the specificity of such a general CSC biomarker as CD44, which is used in the diagnosis of many tumors in cervical cancer, is not sufficiently established. The proto-oncogene c-Kit (protein tyrosine kinase KIT or CD117), a transmembrane cytokine receptor, is phosphorylated and activated by binding to a KIT ligand called stem cell factor, which is a marker of SCSs in ovarian and endometrial cancers and osteosarcoma; however, the proposal that c-Kit is a marker of CSCs in cervical cancer is not yet proved. NANOG is a transcription factor that plays an important role in maintaining the pluripotency of CSCs and regulation of proliferation and asymmetric division; it is used widely as a marker of CSCs that regulate renewal and tumorigenesis in many tumors [63]. Therefore, stem cells play a key role in the physiology of the female reproductive system; however, according to Lopez et al. (2013), this field is still in its infancy—definitive markers need to be identified for more selective isolation and enrichment of stem cells, and further research is needed to assess clinical characteristics, prognosis, survival, and population characteristics; these studies will improve the understanding of carcinogenesis in ovarian and uterine cancer and cervical carcinoma and may be useful in the treatment of these neoplasms [64].

## 10. Markers of Angiogenesis in Cervical Carcinogenesis

The processes of neoangiogenesis and lymphangiogenesis in malignant tumors are associated with growth, invasion, and metastasis [27,66]. During neoangiogenesis, a network of capillaries is formed from the endothelial cells lining small venules, primarily in the tissues adjacent to the tumor; the formed capillary network surrounding the tumor is abnormal in its morphology, density, and vascular permeability [27]. Angiogenesis is stimulated by angiogenic factors, which are prognostic and predictive, the main ones being vascular endothelial growth factor (VEGF), fibroblast growth factor (FGF), angiogenin, transforming growth factor-α (TGF-α). VEGF is an important mediator of neoangiogenesis, stimulating the proliferation of endothelial cells and enhancing vascular permeability, as well as indirectly affecting lymphangiogenesis; VEGF is an important therapeutic target in the development of antiangiogenic anticancer drugs [65,66]. To assess angiogenesis in tumors, antibodies to endothelial cell markers—factor VIII, CD31, CD34, and others—are used. An immunohistochemical study of 234 samples of cervical tissues with antibodies to VEGF and podoplanin (PDPN) with varying degrees of CIN revealed that VEGF expression was significantly stronger in HSIL samples compared to LSIL, and the simultaneous assessment of VEGF and PDPN can provide additional information for possible tactics for treating patients with CIN [66]. In a study of the expression of VEGF-C and VEGF-D, as well as their receptor VEGFR-3, in 152 lesions of the cervix (33 CIN 1, 33 CIN 2, 37 CIN 3, and 49 squamous cell carcinomas), the expression of the VEGFR3 protein was found in more than 50% of lesions in CIN 3 and cervical cancer, compared with 15% in CIN 1 and 2, which indicates that during cervical carcinogenesis, the switch to the lymphangiogenic phenotype may occur at the stage of CIN 3 before the onset of invasion [67].

To assess the relationship between tumor angiogenesis markers and survival, a panel of immunohistochemical factors was used—VEGF, thrombospondin-1 (TSP-1, antiangiogenesis factor), CD31 (a nonspecific endothelial marker), and CD105 (a tumor-specific endothelial marker) [68]. The data obtained during a study of the role of the deletion at locus 4 of pancreatic carcinoma (DPC4) and vascular endothelial growth factor (VEGF) in the development of cervical cancer suggest that the loss of DPC4 and overexpression of VEGF may play an important role in the progression of cervical carcinoma. In cervical cancer, high levels of VEGF expression were found in cervical cancer [69]. VEGF plays an important role in tumor progression by inducing angiogenesis, while as a potent inhibitor of neovascularization, TSP-1 can inhibit angiogenesis in cervical carcinoma. A possible mechanism for tumor progression is the loss of DPC4, which induces angiogenesis by increasing VEGF expression. Apparently, VEGF is a target gene regulated by DPC4, and negative expression of TSP 1 promotes angiogenesis; however, further studies are needed to assess the correlation between DPC4 and TSP 1 [69]. During examination of 44 women with advanced cervical carcinoma, before the beginning of therapy a biopsy and a blood sample were taken from each patient, the density of micro vascularization was assessed in the biopsy material using antibodies to CD34, and the expression of angiogenic factors VEGFR, EGFR, and COX-2 was determined. It was found that the response to radiotherapy and chemotherapy in cervical cancer is associated with clinical and molecular factors, such as tumor size, VEGFR2 expression, mRNA level, and patient age.

Expression of VEGFR2 is a prognostic factor in the development of cervical cancer, but further studies are needed to assess the relationships between various biomarkers [70]. When evaluating the predictive value of vimentin, TP53, and podoplanin in patients with cervical cancer, it was found that the levels of mRNA expression of vimentin, TP53, and podoplanin were significantly increased in the carcinoma tissue compared to adjacent normal cervical tissues; the expression of vimentin, TP53 and podoplanin was found to be correlated with the survival of patients with cervical cancer, which indicates their role as valuable biomarkers for the diagnosis and treatment of cervical cancer [71]. Thus, modern data indicate the important prognostic and predictive value of factors regulating the processes of neoangiogenesis and lymphangiogenesis in cervical carcinoma; however, further research is needed to understand the role of individual factors and their interrelationships.

## 11. Vaginal Microbiome, Inflammation, and Immune Homeostasis

Currently, several studies have investigated the role of the vaginal microbiome in the progression of HPV infection of the cervix [56,72,73,74,75,76,77,78]. New data are emerging that indicate a possible role for increased diversity of the vaginal microbiota, in combination with a reduced relative quantity of *Lactobacillus* spp. in cases of persistent HPV, in the development of precancerous lesions and cervical cancer [72]. A meta-analysis to assess the relationship between vaginal microbiota composition, human papillomavirus (HPV) infection, and progression of cervical dysplasia and cancer found that vaginal microbiota with predominance of non-*Lactobacilli* or *Lactobacillus iners* species was associated with a higher chance of presence (3–5-fold increase) of any common HPV type and a higher frequency (2–3-fold increase) of high-risk HPV and cervical dysplasia/cancer in comparison with *Lactobacillus crispatus* [73]. The results of this meta-analysis support an association between certain types of bacterial community in the vaginal microbiota and HPV infection, as well as HPV-related diseases, which may be useful in defining treatment or as a biomarker of HPV-related disease. A meta-analysis to identify a possible association between bacterial vaginosis and the development of CIN provided evidence for a positive association [74]. The cervicovaginal microbiota is capable of modulating immune responses, and it has recently been found that there is a link between the state of the microbiota and the risk of persistent HPV infection [72,73]. The role of the microbial vaginal environment in the processes of oncogenesis in cervical cancer is currently very important, and the state of the microbiome can be considered to be a biomarker for lesions of the cervix and for identifying women with a high risk of persistence of HPV, CIN, and cervical cancer [75,76,77,78].

In addition to infection caused by the HPV, immune homeostasis in the genital tract is important in cervical carcinogenesis. Recent studies have shown that HPV-infected cells contribute to chronic inflammation of the stroma and interact with the local immune microenvironment, and that chronic inflammation and disorders of the local immune microenvironment play a crucial role in the progression from precancerous lesions to invasive cancer [79]. When analyzing the background processes in patients with various degrees of neoplasia, it was found that most often there were inflammatory processes of the cervix, often in combination with cervical ectopia; in severe dysplasia, inflammation was diagnosed in 62.1% of cases; in non-invasive cancer, in 54%; and in the cases with invasive cancer, in 73.9% [80]. Understanding of the immunological mechanisms promoting the development of HPV-associated cancer is not only likely to contribute to a more accurate diagnosis of the progression of precancerous lesions but also necessary for creation of new immunotherapeutic approaches [79]. Thus, the role of vaginal microbiome immune mechanisms in the initiation and progression of neoplastic lesions of the cervix, as well as many another aspects of cervical cancer carcinogenesis, has yet to be fully elucidated.

The concept of stages of cervical carcinogenesis was originally based on pathomorphological changes; it has been confirmed by studies using genome-wide screening of molecular disorders at different phases of cervical cancer development. Each molecular profile (genome, transcriptome, proteome, etc.) in the dynamics of the development of the disease progression gradually accumulates multiple aberrations and thus contributes to the development of the tumor [81]. Despite the large number of publications dedicated to various aspects of cervical carcinogenesis, pathologists still play an important role in the development of diagnostic approaches and targeted therapies aimed at tumors characterized by a certain phenotype and the presence of biomarkers that allow the response to targeted therapy to be predicted, enabling the monitoring of response to treatment [92].

## 12. Conclusions

The concept of stages of cervical carcinogenesis was originally based on pathomorphological changes; it was confirmed by studies using genome-wide screening of molecular disorders at different phases of cervical cancer development. Each molecular profile (genome, transcriptome, proteome, etc.) in the dynamics of the development of the disease progression gradually accumulates multiple aberrations and thus contributes to the development of the tumor [81]. Despite the large number of publications dedicated to various aspects of cervical carcinogenesis, pathologists still play an important role in the development of diagnostic approaches and targeted therapies aimed at tumors characterized by a certain phenotype and the presence of biomarkers that allow the response to targeted therapy to be predicted, enabling the monitoring of response to treatment [92]. It should be emphasized the high importance of modern screening studies in various countries aimed at detecting neoplasms of the cervix using such well-known markers as P16 and Ki-67 [93,94,95,96]. Immunohistochemistry is used to study both the various stages of carcinogenesis, but also to search for effective prognostic and predictive factors. Thus, immunohistochemical markers of apoptosis p53 and human epidermal growth factor receptor 2 protein levels were evaluated by as potential prognostic factors in cervical cancer associated with a poor prognosis [97]. At present, molecular, biochemical and genetic aspects of cervical carcinogenesis continue to be investigated [98,99]. PI3K/Akt, Wnt/β-catenin, ERK/MAPK, NF-κB, YY1, AP-1, JAK/STAT and CXCL12/CXCR4 signaling pathways have a significant role in the cervical cancerogenesis in HPV-infected individuals [98]. The modern methods are used to search for serum markers in cervical cancer by mean of perspective technologies such as magnetic bead-based weak cation—exchange chromatography fractionation combined with matrix-assisted laser desorption / ionization-time of flight mass spectrometry [100], the multiplex proximity extension assay is used [101]. A significant amount of modern research of cervical neoplasia is aimed at identifying genetic, epigenetic changes, revealing the possible role of long non-coding RNAs and circular RNAs in cervical carcinogenesis [102,103,104,105,106,107]. Modern tendencies in the problem of cervical neoplasia include research on immune factors and the vaginal microbiome [108,109,110]. Thus, to study various aspects of cervical carcinogenesis, various methods are currently used, both traditional cytological and modern immunohistochemical, molecular, genetic and others; multidisciplinary approaches seem to be promising.

Numerous scientific publications devoted to various aspects of neoplasia progression, prognosis, and treatment of cervical cancer indicate that many issues of cervical carcinogenesis are currently insufficiently studied and not fully understood. Despite the avalanche of new data in the field of cervical oncobiology, many issues remain to be resolved in the future, apparently with the help of modern multidisciplinary approaches, and the problem of finding effective biomarkers for differential diagnosis and targets for multipurpose therapy of cervical neoplasms remains extremely urgent.

## Figures and Tables

**Table 1:** Biomarkers for investigation of carcinogenesis, precancerous lesions and cervical carcinoma.

Croups of Markers	Markers	References
Biomarkers of HPV infection and carcinogenesis	HPV DNA, E6/E7 mRNA p53, Rb, p16INK4a, telomerase RNA gene (TERC), serum SCC-Ag, OCVA1	[8,10,11,12,13,15,20,21,22,23,24,25,26]
Markers of cell cycle and proliferation	Ki-67, cyclin D1, p53, p63	[12,26,27,28,29,30,31,32,33,34,35,36]
Markers of apoptosis	P53, BCL-2, BCL-XL, BAX	[10,27,37,38,39,40,41,42,43,44]
Expression of cytokeratins–markers of differentiation	CK7, CK8, CK17, CK19	[27,36,37,45,46,47,48,49]
Markers of cell adhesion, invasion and metastasis	E-cadherin, P-cadherin, CD44, ADAM9, MT1-MMP, TIMP-1, TIMP-2, MT1-MMP, MMP-2, MMP-1, MMP-9, MMP-14, proMMP-14 furin, gelatinase, TIMP-1 and TIMP-2	[27,50,51,52,53,54,55]
Biomarkers of cancer stem cells	Nanog, nucleostemin (NS), musashi1 (Msi1), SOX2, KLF4, CD133, Cd44, ALDH1, CD49f, ABCG2, BMI1, PIWIL2, LGR5, OCT4, CD117	[48,56,57,58,59,60,61,62,63,64]
Markers of angiogenesis	VEGF, podoplanin (PDPN), thrombospondin-1 (TSP-1, antiangiogenesis factor), CD31 (a nonspecific endothelial marker), CD34, CD105 (a tumor-specific endothelial marker)	[27,65,66,67,68,69,70,71]
Vaginal microbiome, inflammation and immune homeostasis	Evaluation of the diversity of cervicovaginal microbiome	[56,72,73,74,75,76,77,78,79,80,81]
